# Supplementary figures and images for: DNALI1 interacts with the MEIG1/PACRG complex within the manchette and is required for proper sperm flagellum assembly in mice
Source: eLife. 2023 Apr 21;12:e79620. doi: 10.7554/eLife.79620 (PMC10185345; doi:10.7554/eLife.79620)

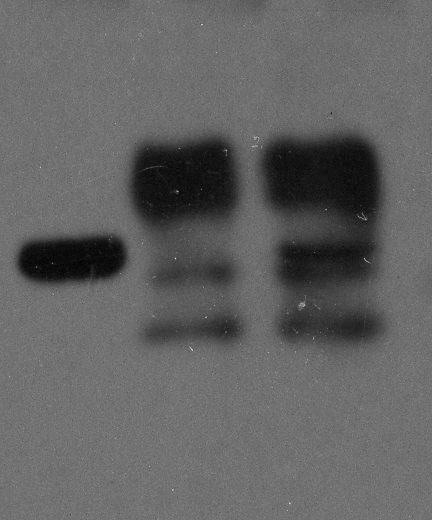

Supplement: Figure 1—source data 1. [file elife-79620-fig1-data1.zip › Figure 1 source data 1 /Fig 1 co-ip myc.jpg]

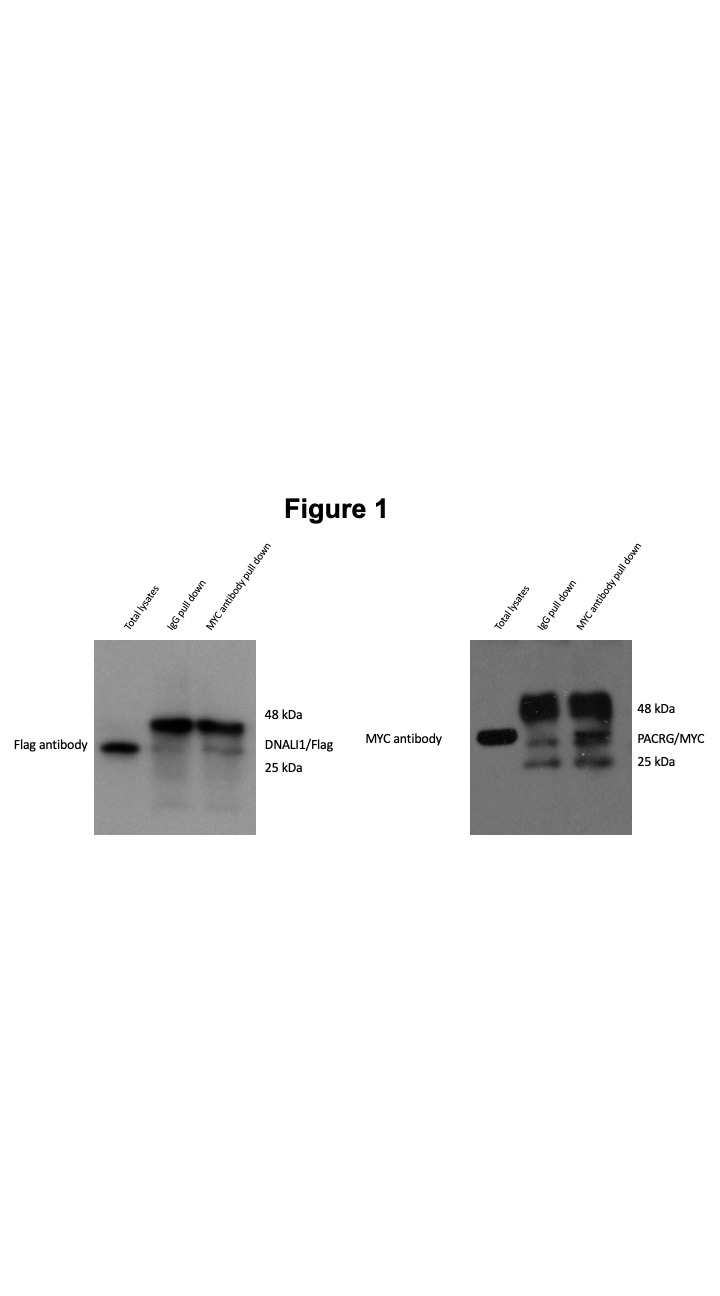

Supplement: Figure 1—source data 1. [file elife-79620-fig1-data1.zip › Figure 1 source data 1 /Figure 1 - source data 1.jpeg]

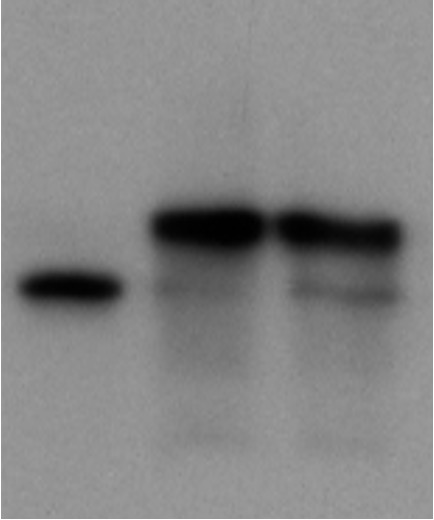

Supplement: Figure 1—source data 1. [file elife-79620-fig1-data1.zip › Figure 1 source data 1 /Fig 1 co-ip flag.jpg]

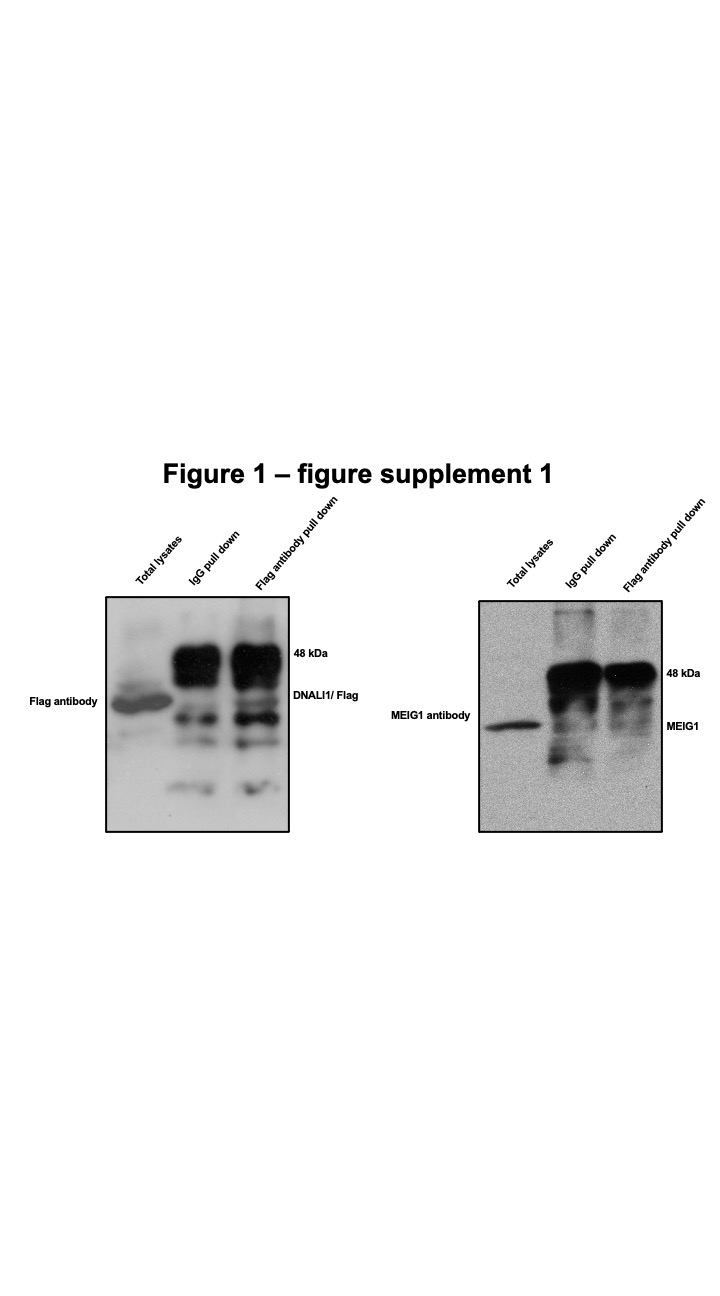

Supplement: Figure 1—figure supplement 1—source data 1. [file elife-79620-fig1-figsupp1-data1.zip › Figure 1 figure supplement 1 source data 1/Figure 1 - figure supplement 1 - source data 1.jpeg]

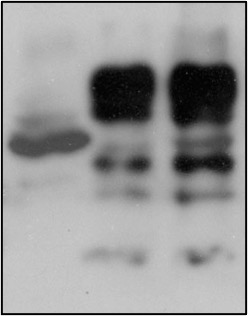

Supplement: Figure 1—figure supplement 1—source data 1. [file elife-79620-fig1-figsupp1-data1.zip › Figure 1 figure supplement 1 source data 1/fig 1 supp 1 source data 1.jpg]

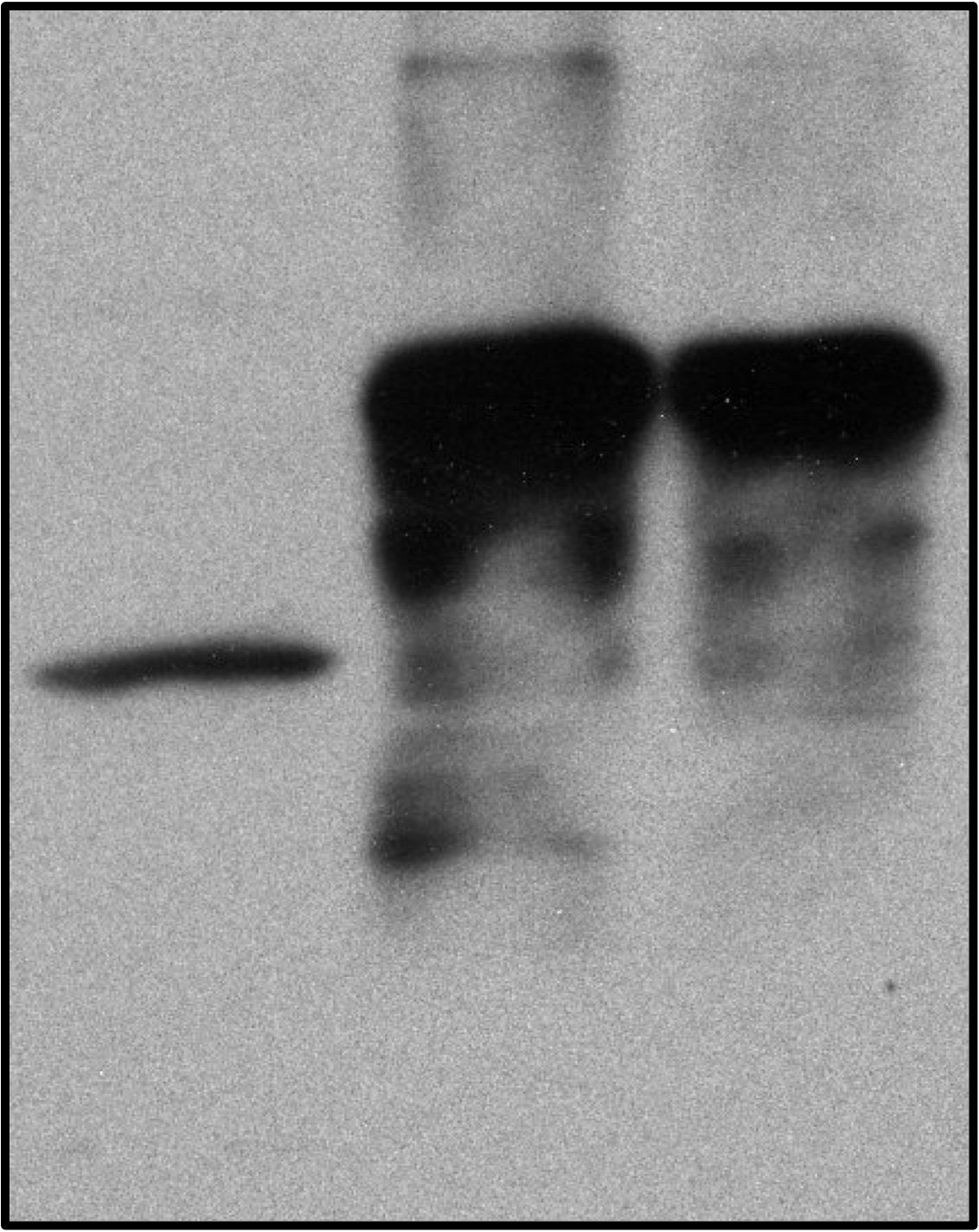

Supplement: Figure 1—figure supplement 1—source data 1. [file elife-79620-fig1-figsupp1-data1.zip › Figure 1 figure supplement 1 source data 1/fig 1 supp 1 data source 2.jpg]

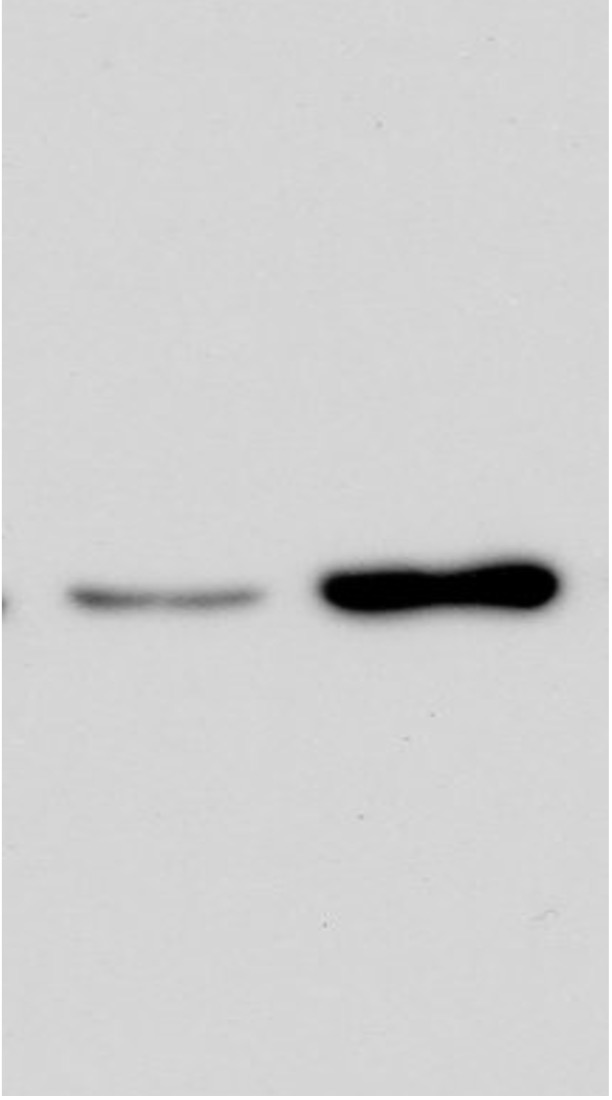

Supplement: Figure 2—source data 1. [file elife-79620-fig2-data1.zip › Figure 2 source data 1/fig2b his femto.jpg]

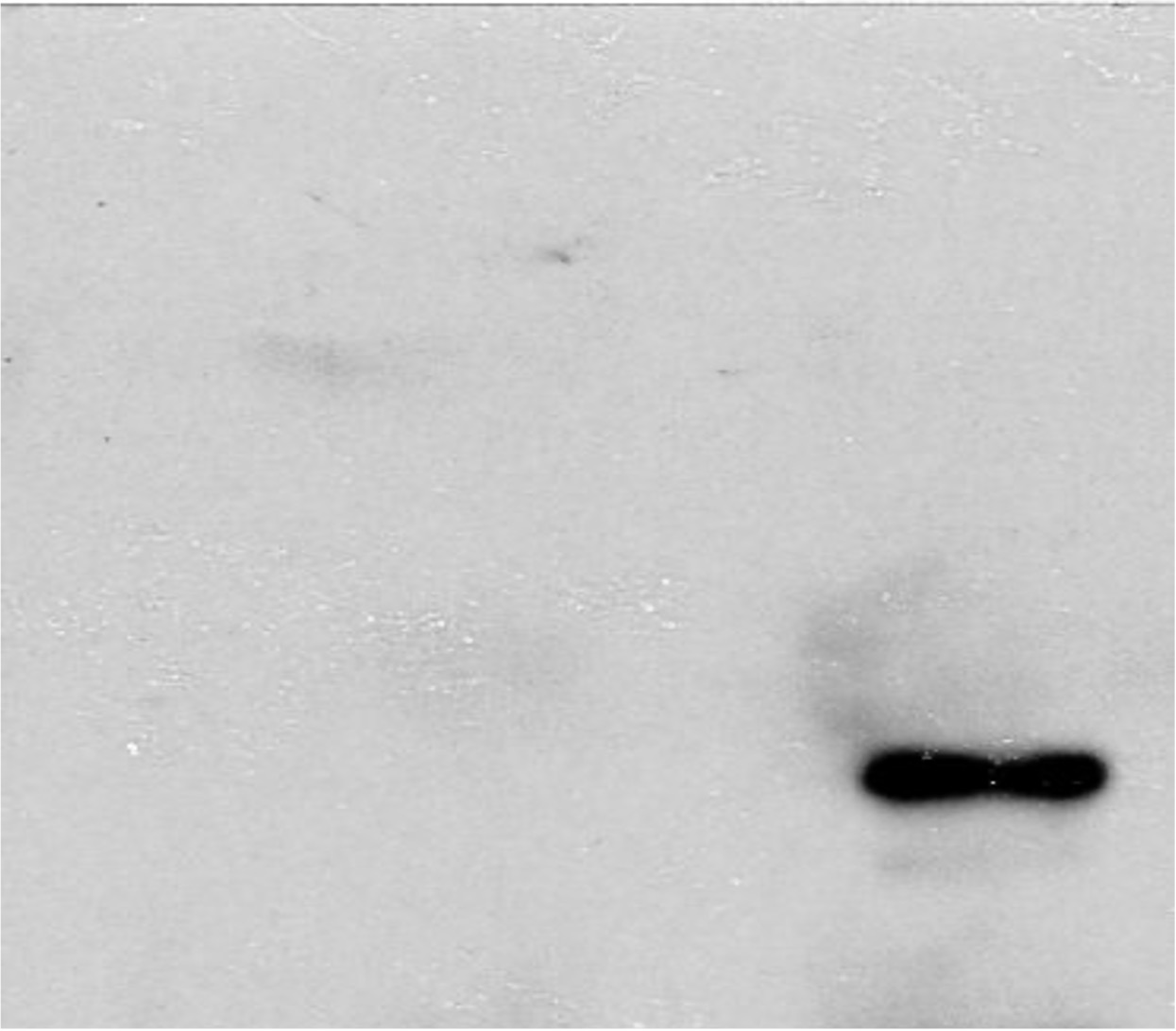

Supplement: Figure 2—source data 1. [file elife-79620-fig2-data1.zip › Figure 2 source data 1/fig2b dnali1.jpg]

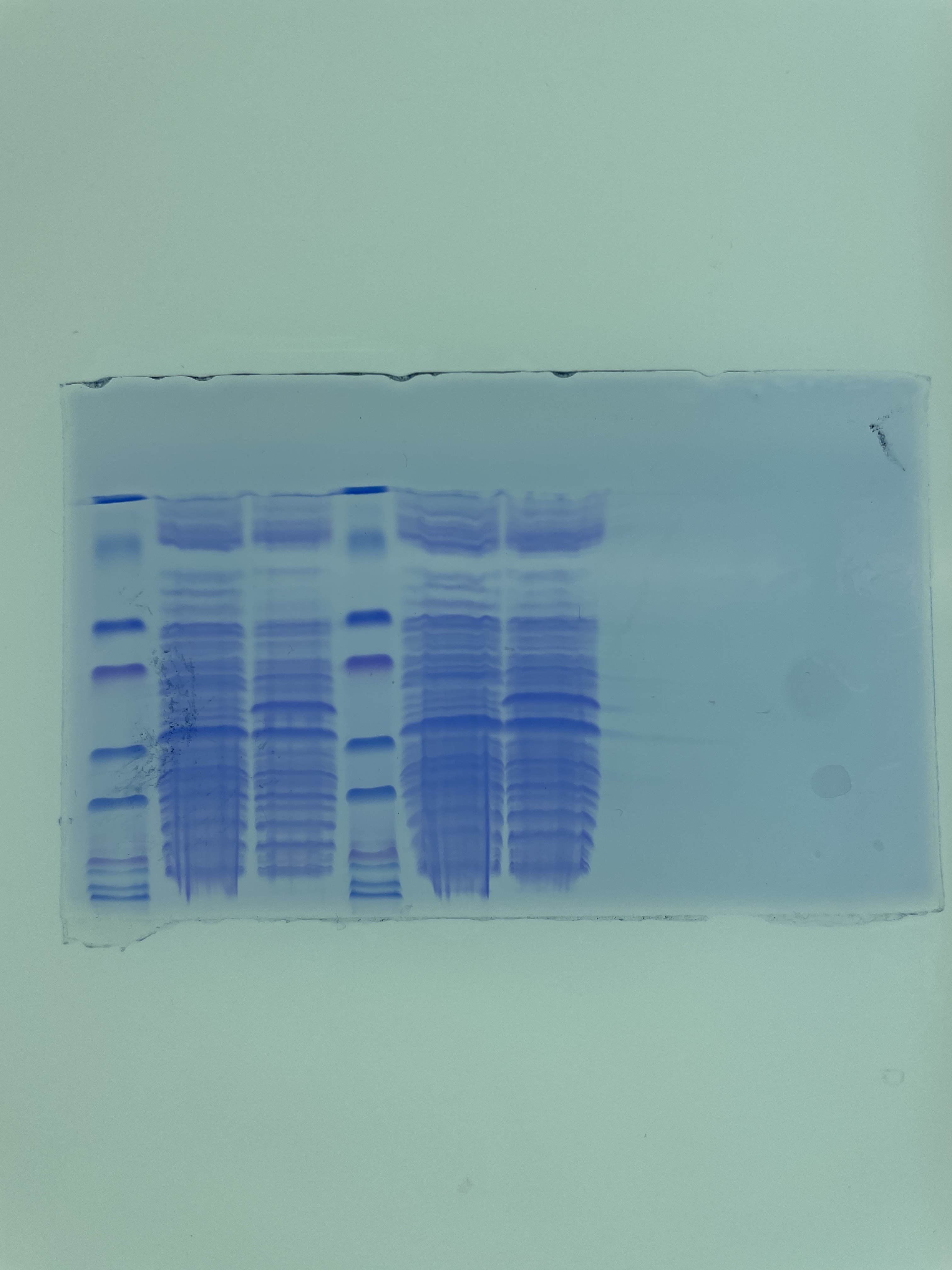

Supplement: Figure 2—source data 1. [file elife-79620-fig2-data1.zip › Figure 2 source data 1/fig 2b gel.jpg]

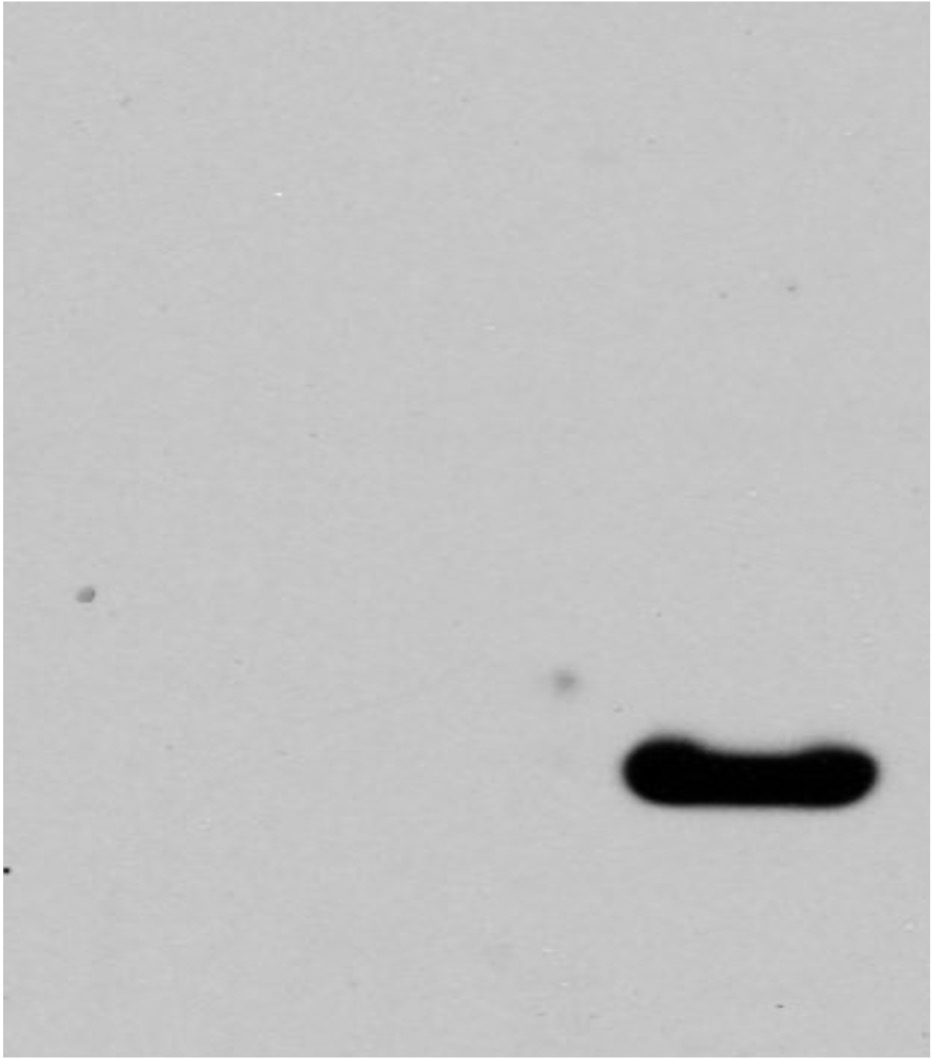

Supplement: Figure 2—source data 1. [file elife-79620-fig2-data1.zip › Figure 2 source data 1/fig2a myc.jpg]

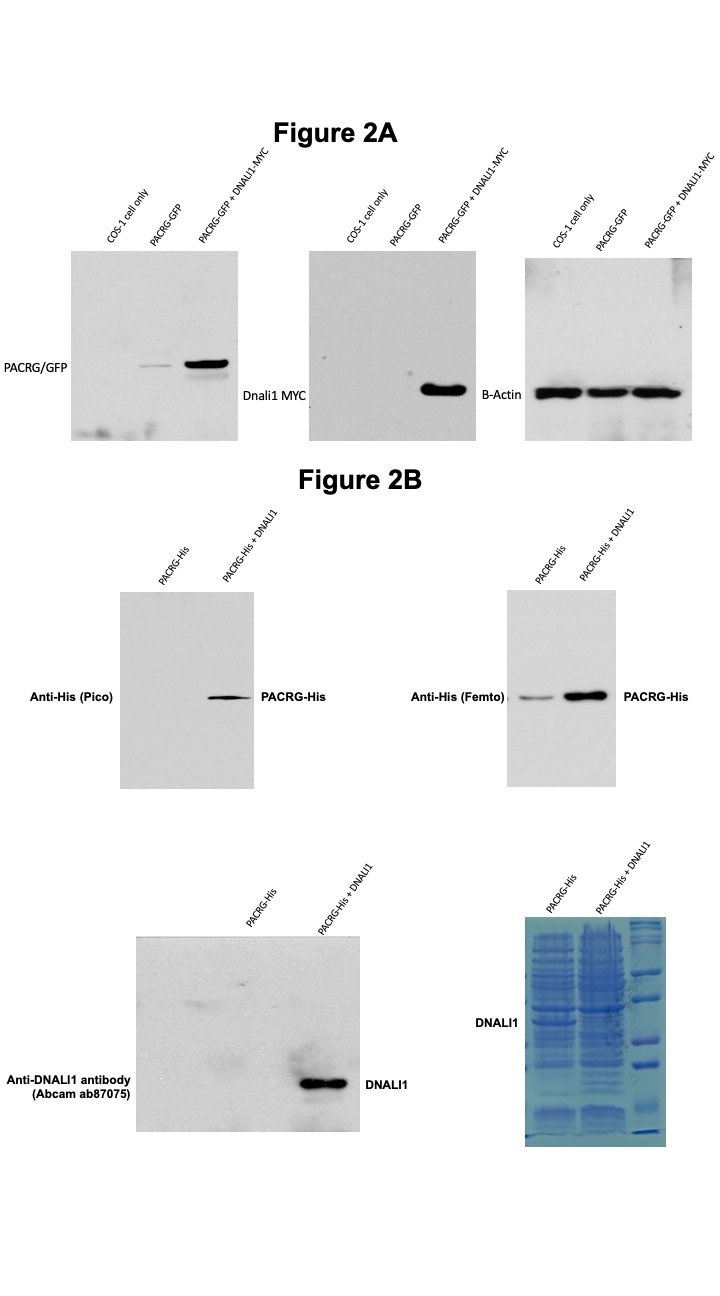

Supplement: Figure 2—source data 1. [file elife-79620-fig2-data1.zip › Figure 2 source data 1/Figure 2 - source data 1.jpeg]

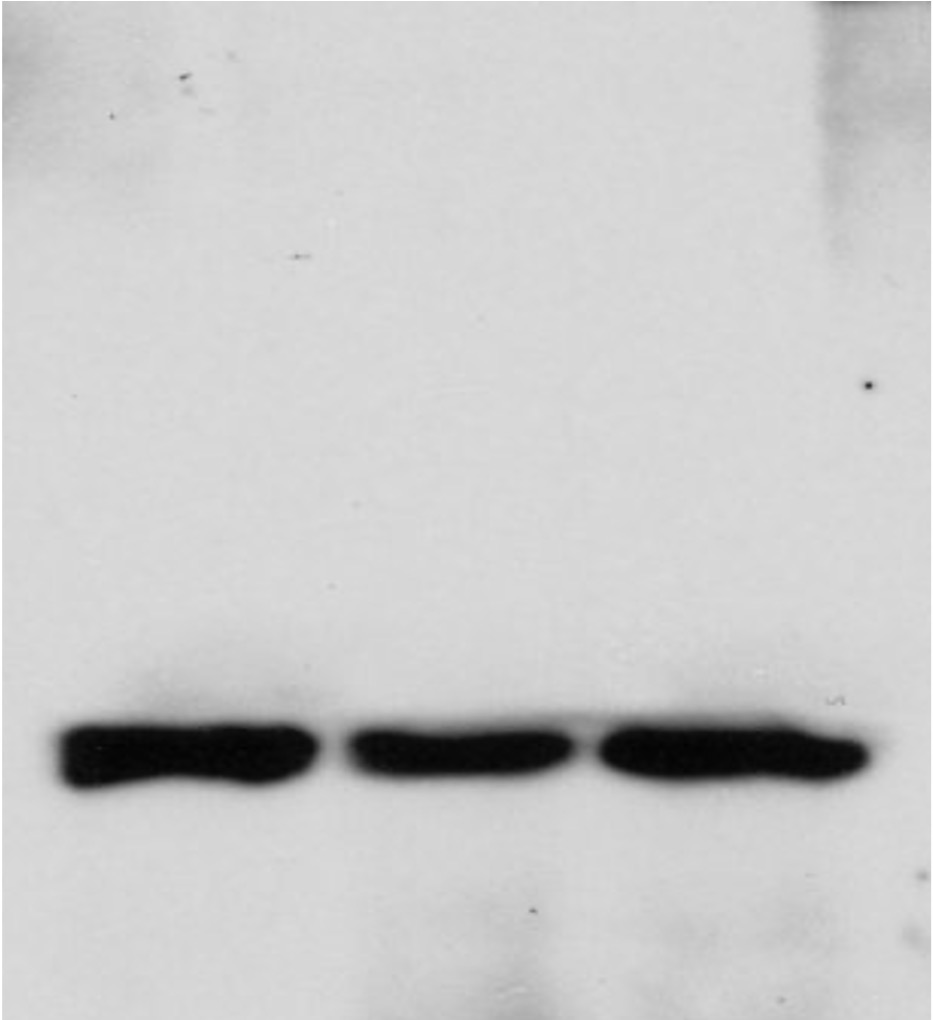

Supplement: Figure 2—source data 1. [file elife-79620-fig2-data1.zip › Figure 2 source data 1/fig2a bactin.jpg]

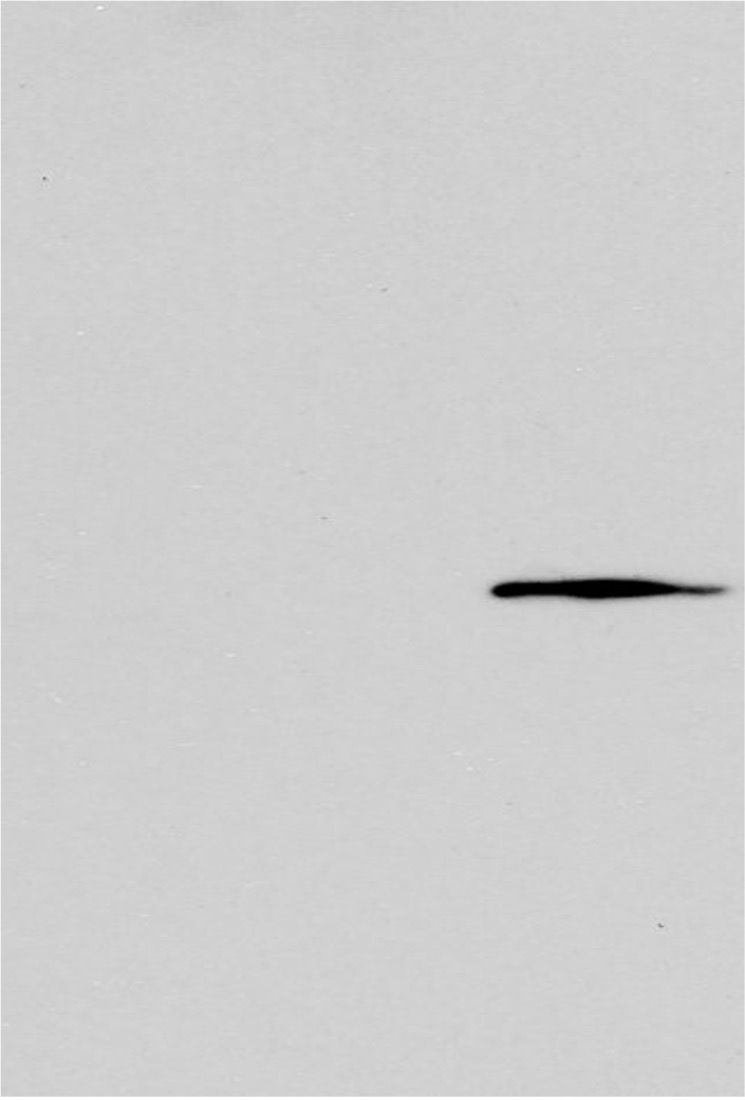

Supplement: Figure 2—source data 1. [file elife-79620-fig2-data1.zip › Figure 2 source data 1/fig2b his pico.jpg]

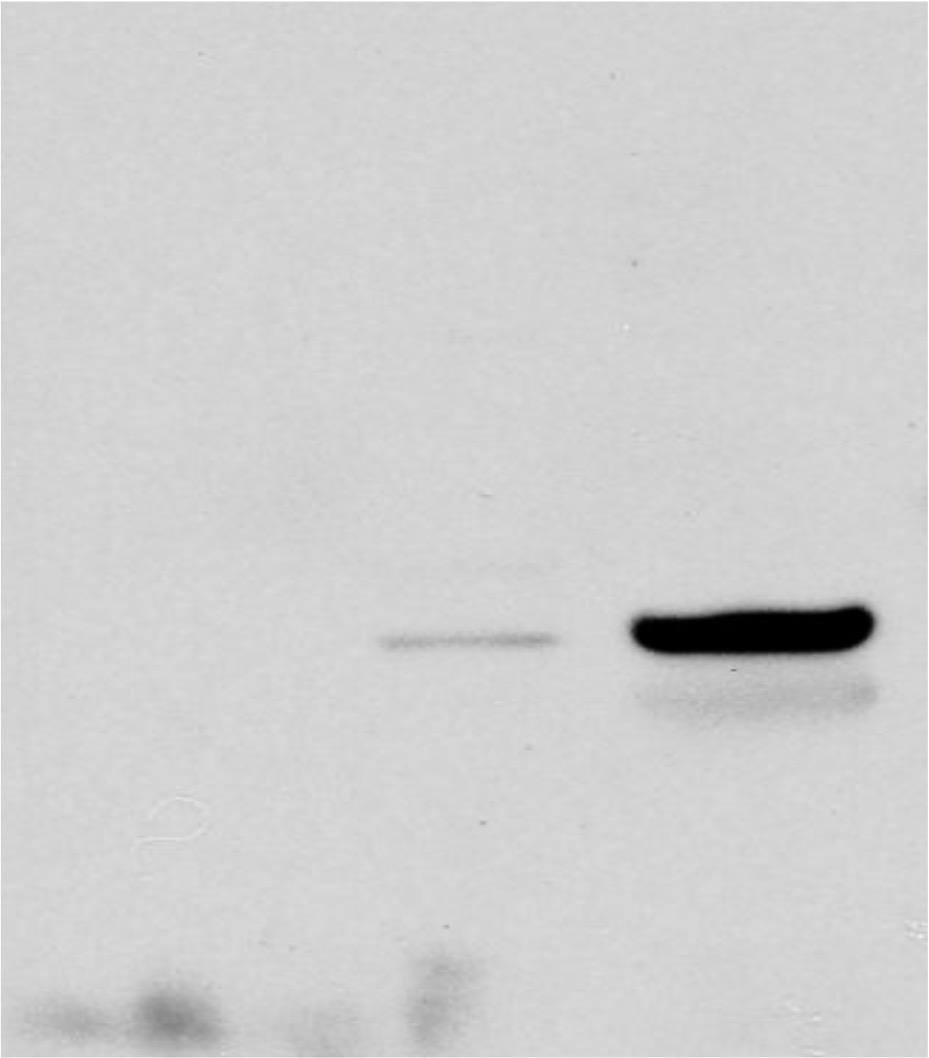

Supplement: Figure 2—source data 1. [file elife-79620-fig2-data1.zip › Figure 2 source data 1/fig2a gfp.jpg]

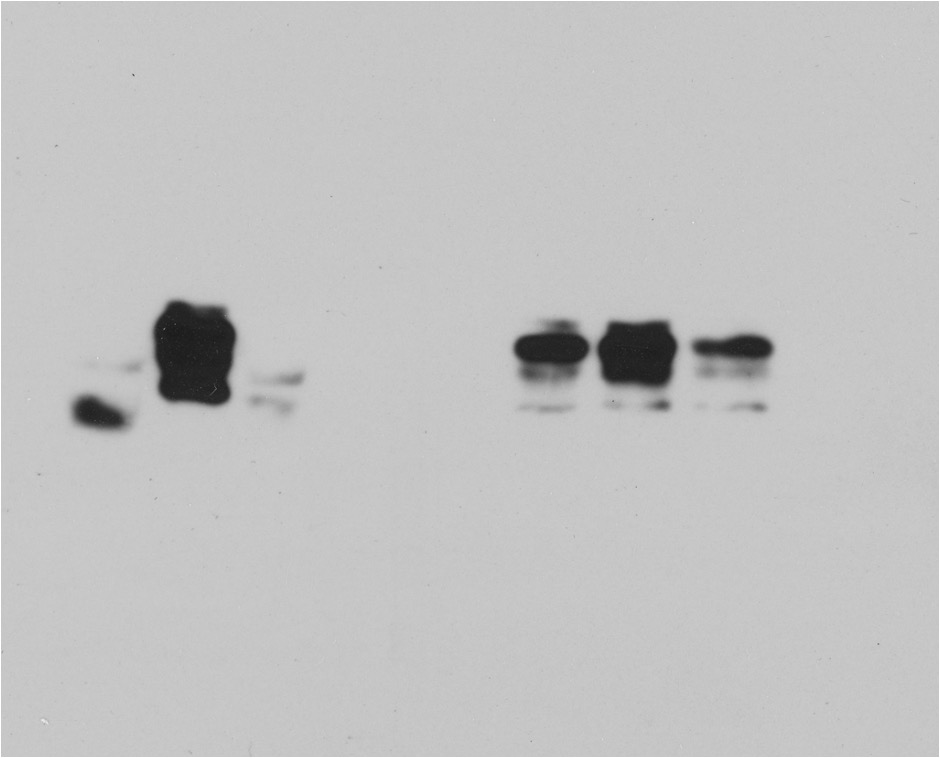

Supplement: Figure 3—source data 1. [file elife-79620-fig3-data1.zip › Figure 3 source data 1/fig 3 anti dnali1.jpg]

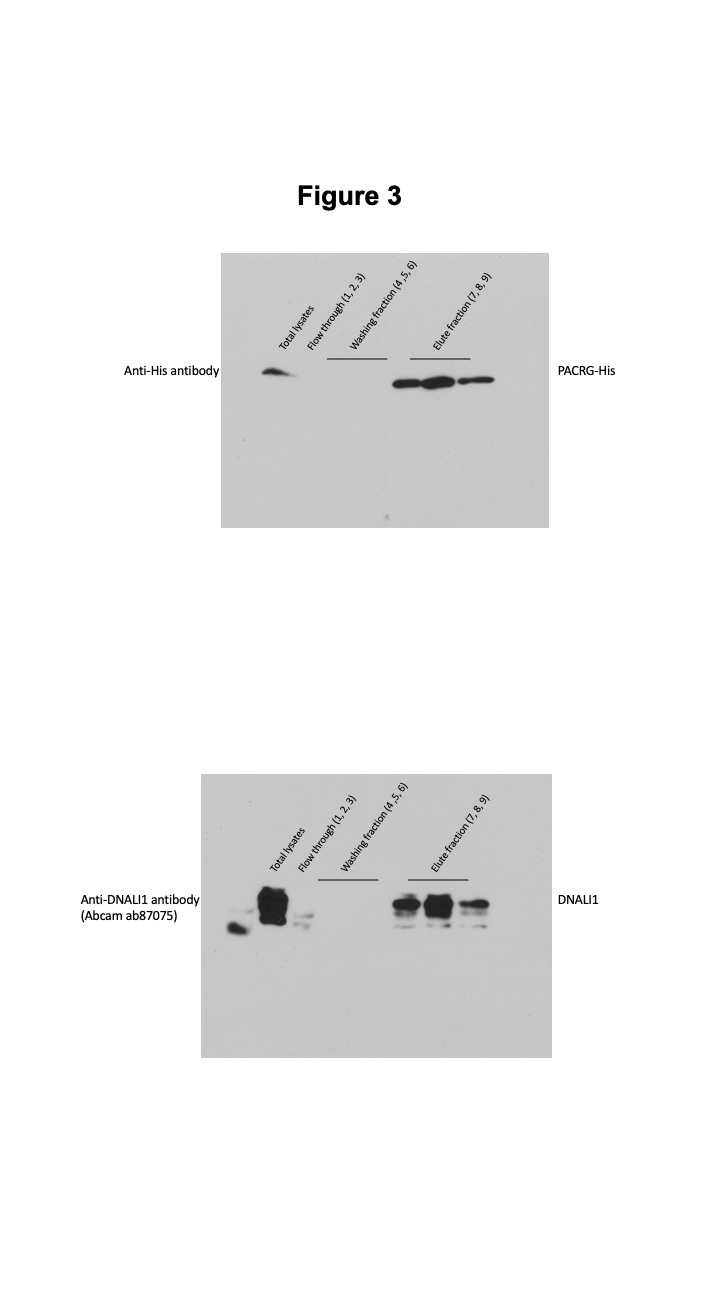

Supplement: Figure 3—source data 1. [file elife-79620-fig3-data1.zip › Figure 3 source data 1/FIgure 3 - source data 1.jpeg]

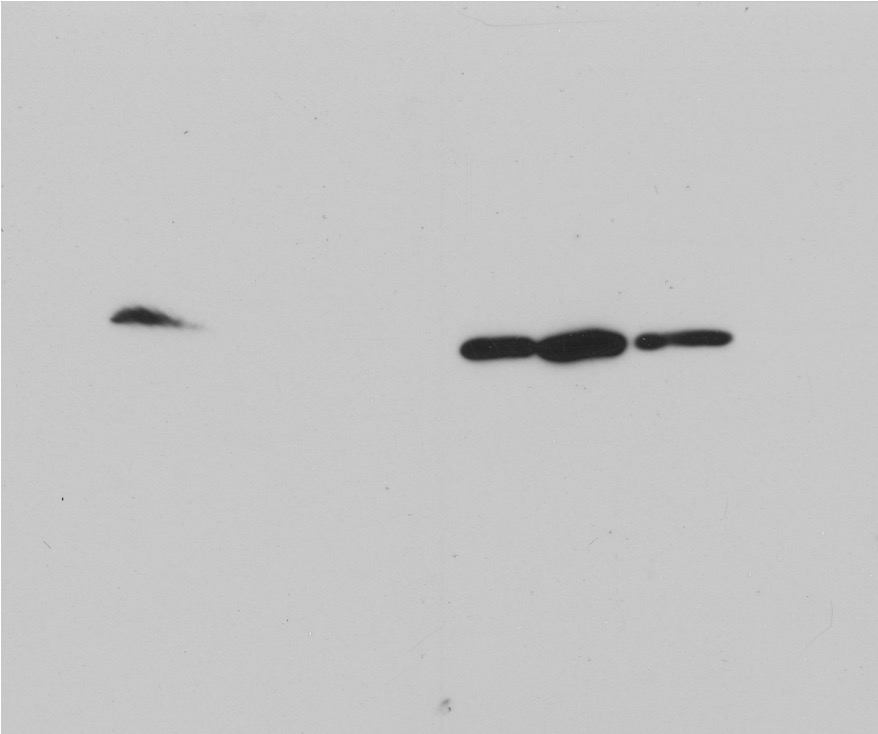

Supplement: Figure 3—source data 1. [file elife-79620-fig3-data1.zip › Figure 3 source data 1/fig 3 anti-His.jpg]

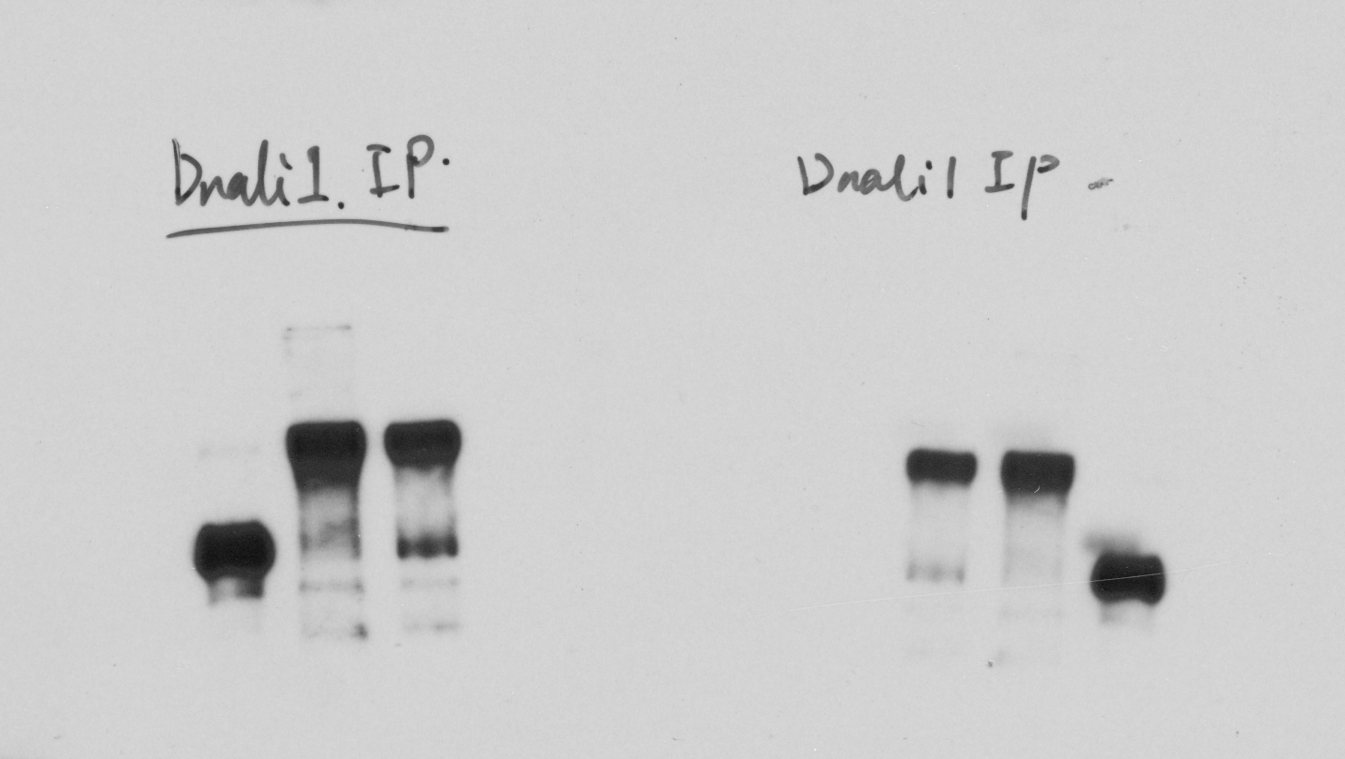

Supplement: Figure 4—source data 1. [file elife-79620-fig4-data1.zip › Figure 4 source data 1/figure 4 source data 1.jpg]

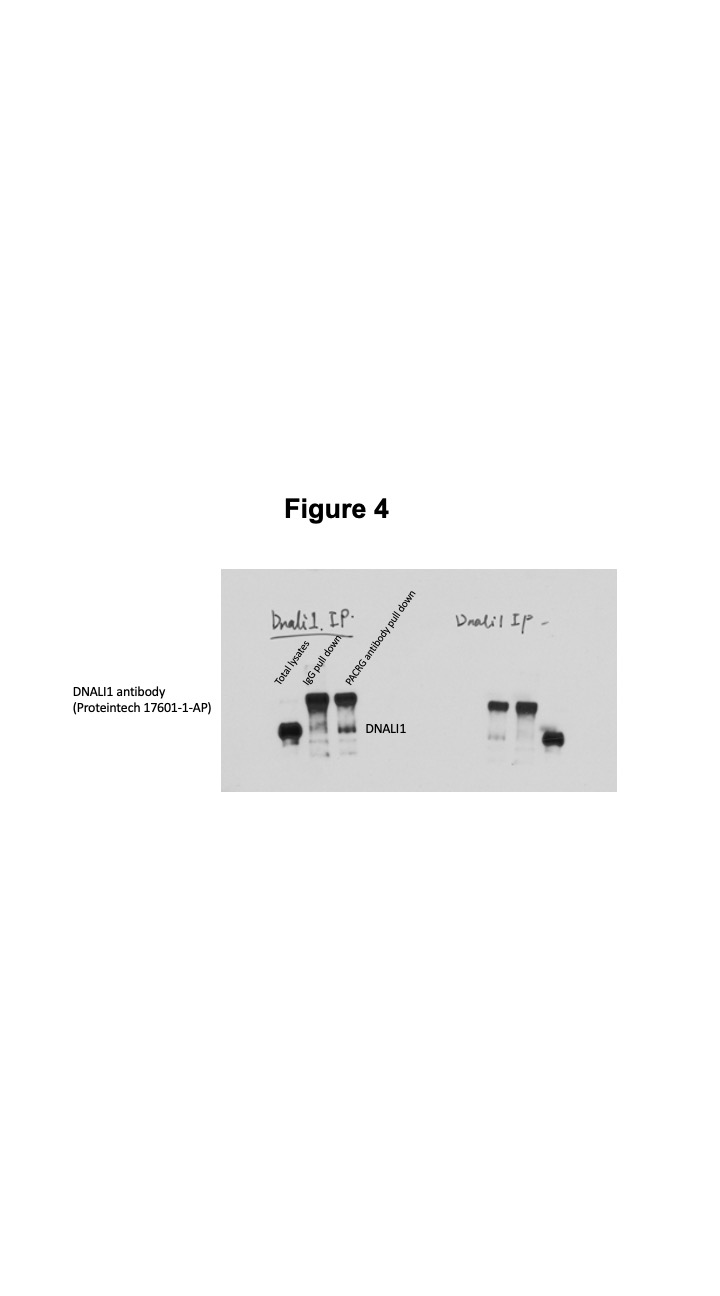

Supplement: Figure 4—source data 1. [file elife-79620-fig4-data1.zip › Figure 4 source data 1/Figure 4 - source data 1/Figure 4 - source data 1.jpeg]

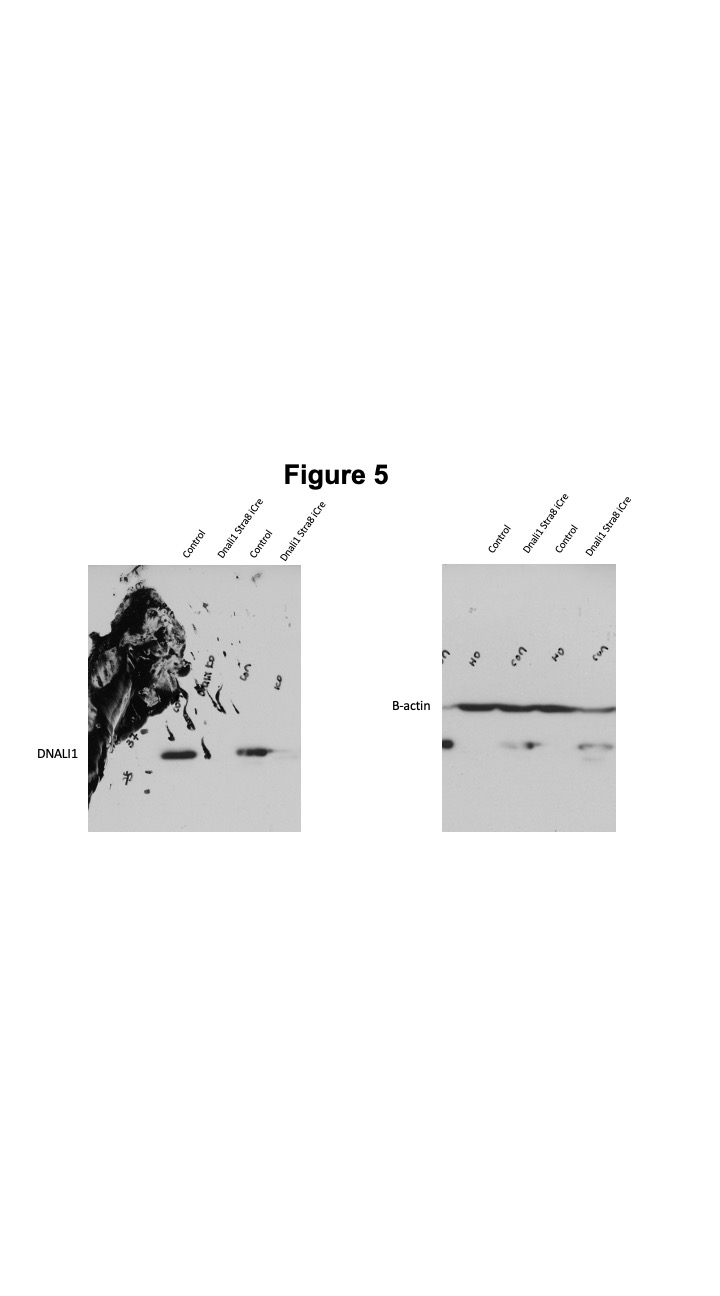

Supplement: Figure 5—source data 1. [file elife-79620-fig5-data1.zip › Figure 5 source data 1/Figure 5 - source data 1.jpeg]

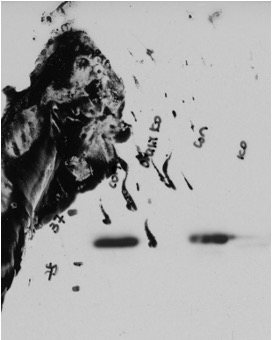

Supplement: Figure 5—source data 1. [file elife-79620-fig5-data1.zip › Figure 5 source data 1/fig5a dnali1.jpg]

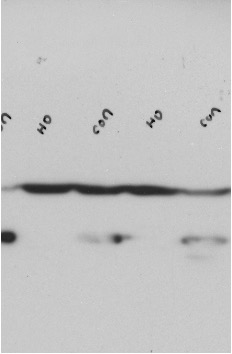

Supplement: Figure 5—source data 1. [file elife-79620-fig5-data1.zip › Figure 5 source data 1/fig5a bactin.jpg]

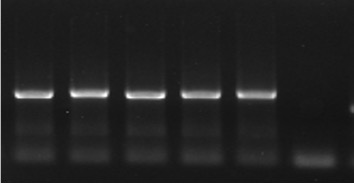

Supplement: Figure 5—figure supplement 1—source data 1. — Upper panel: primer set to analyze COIN Dnali1 allele (844 bp); middle panel: primer set to analyze the wild-type allele (129 bp); lower panel: primer set to detect Cre. [file elife-79620-fig5-figsupp1-data1.zip › Figure 5 figure supplement 1 source data 1/dnali1 COIN.jpg]

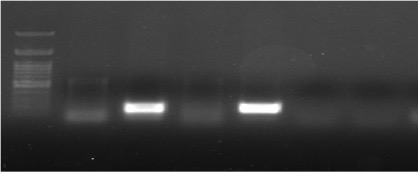

Supplement: Figure 5—figure supplement 1—source data 1. — Upper panel: primer set to analyze COIN Dnali1 allele (844 bp); middle panel: primer set to analyze the wild-type allele (129 bp); lower panel: primer set to detect Cre. [file elife-79620-fig5-figsupp1-data1.zip › Figure 5 figure supplement 1 source data 1/wild type.jpg]

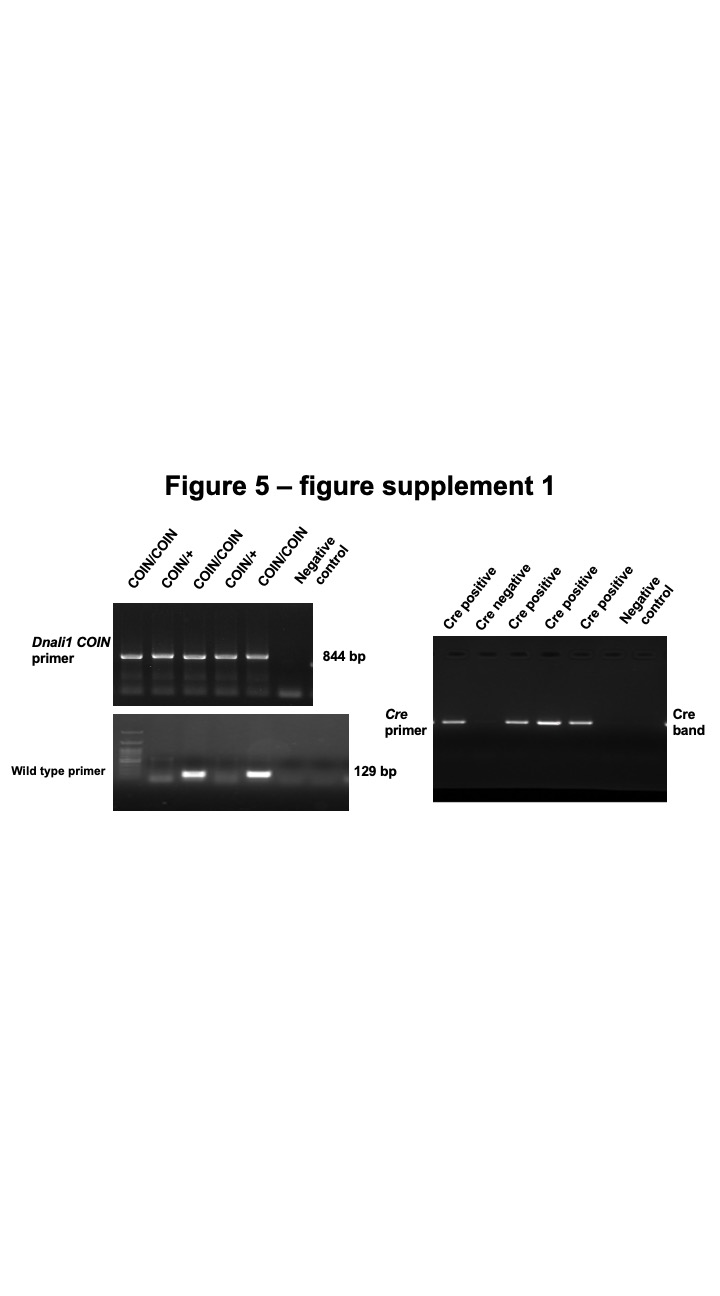

Supplement: Figure 5—figure supplement 1—source data 1. — Upper panel: primer set to analyze COIN Dnali1 allele (844 bp); middle panel: primer set to analyze the wild-type allele (129 bp); lower panel: primer set to detect Cre. [file elife-79620-fig5-figsupp1-data1.zip › Figure 5 figure supplement 1 source data 1/Figure 5 - figure supplement 1 - source data 1.jpeg]

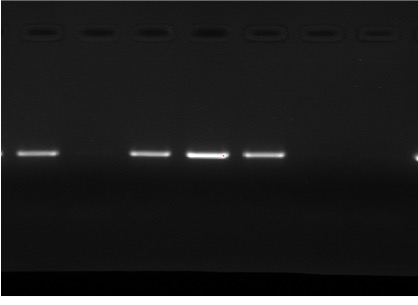

Supplement: Figure 5—figure supplement 1—source data 1. — Upper panel: primer set to analyze COIN Dnali1 allele (844 bp); middle panel: primer set to analyze the wild-type allele (129 bp); lower panel: primer set to detect Cre. [file elife-79620-fig5-figsupp1-data1.zip › Figure 5 figure supplement 1 source data 1/cre.jpg]

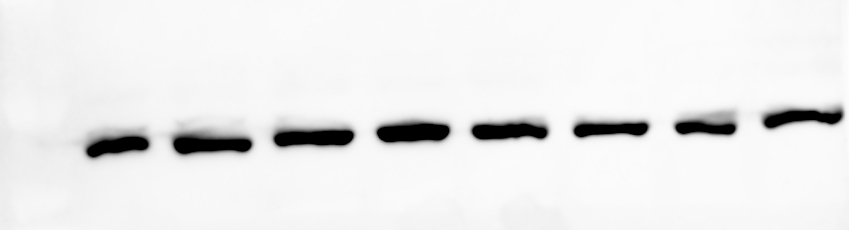

Supplement: Figure 10—source data 1. [file elife-79620-fig10-data1.zip › Figure 10 source data 1/Pacrg.png]

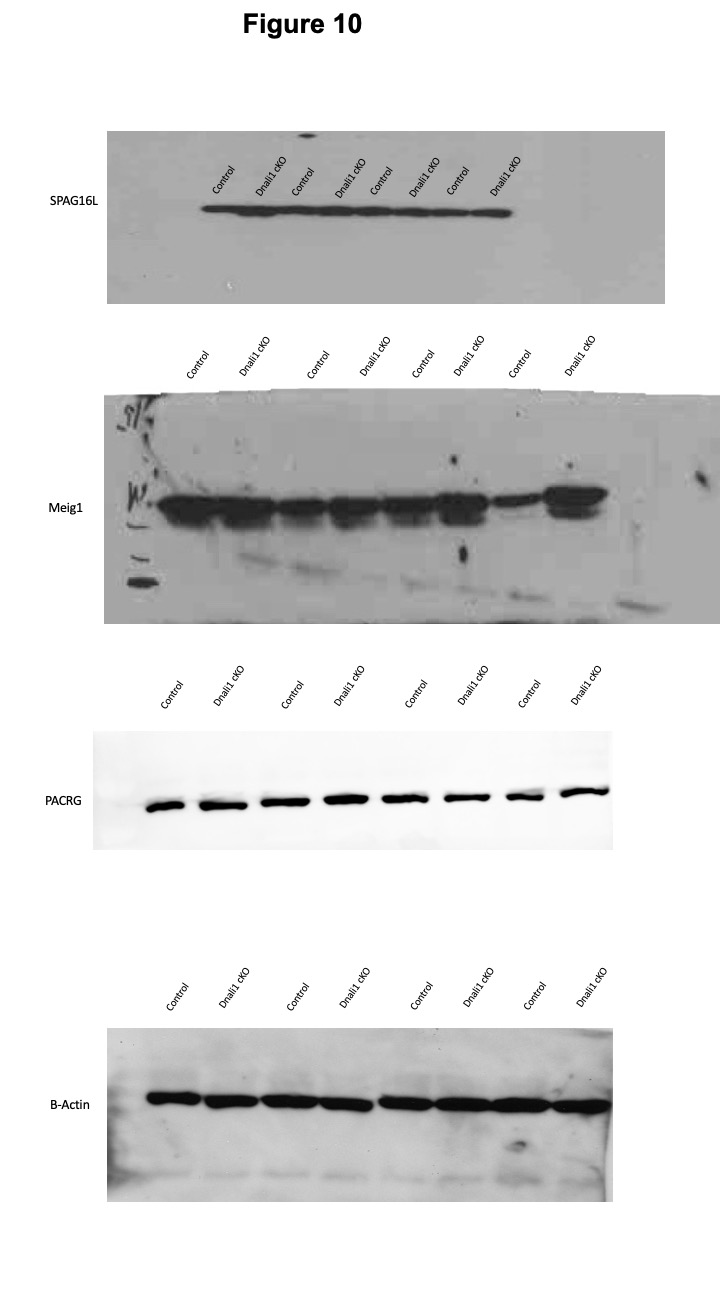

Supplement: Figure 10—source data 1. [file elife-79620-fig10-data1.zip › Figure 10 source data 1/Figure 10 source data annotated.jpg]

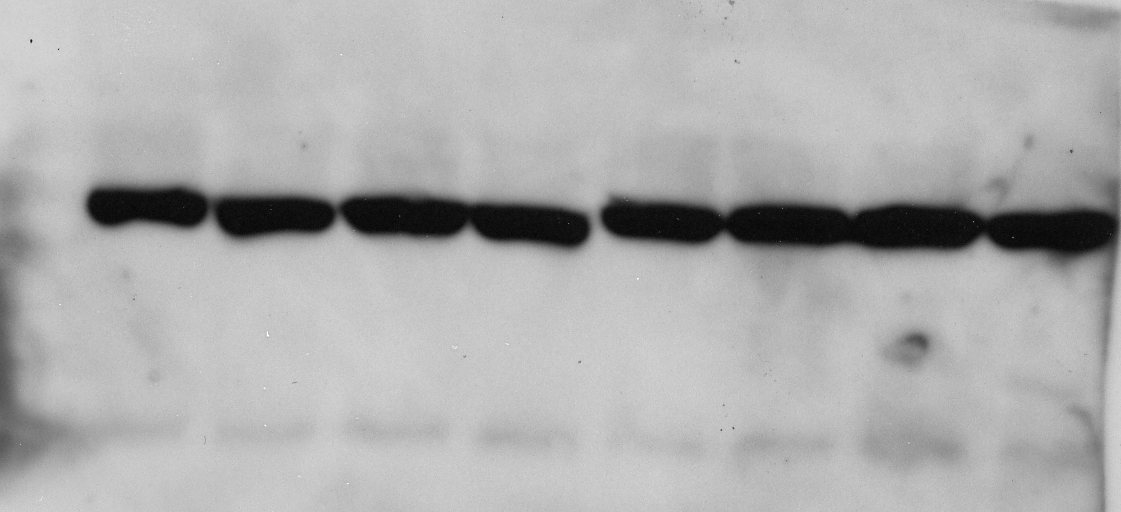

Supplement: Figure 10—source data 1. [file elife-79620-fig10-data1.zip › Figure 10 source data 1/Bactin001.jpg]

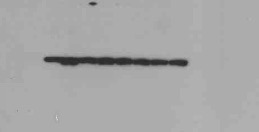

Supplement: Figure 10—source data 1. [file elife-79620-fig10-data1.zip › Figure 10 source data 1/spag16L.jpg]

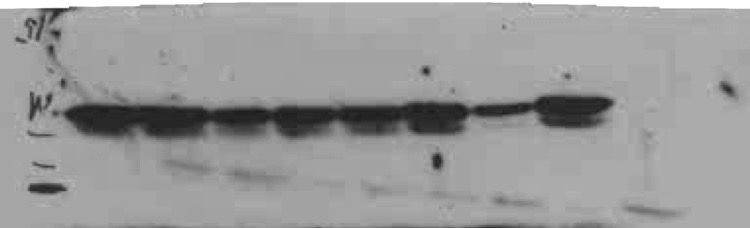

Supplement: Figure 10—source data 1. [file elife-79620-fig10-data1.zip › Figure 10 source data 1/Meig1.jpg]

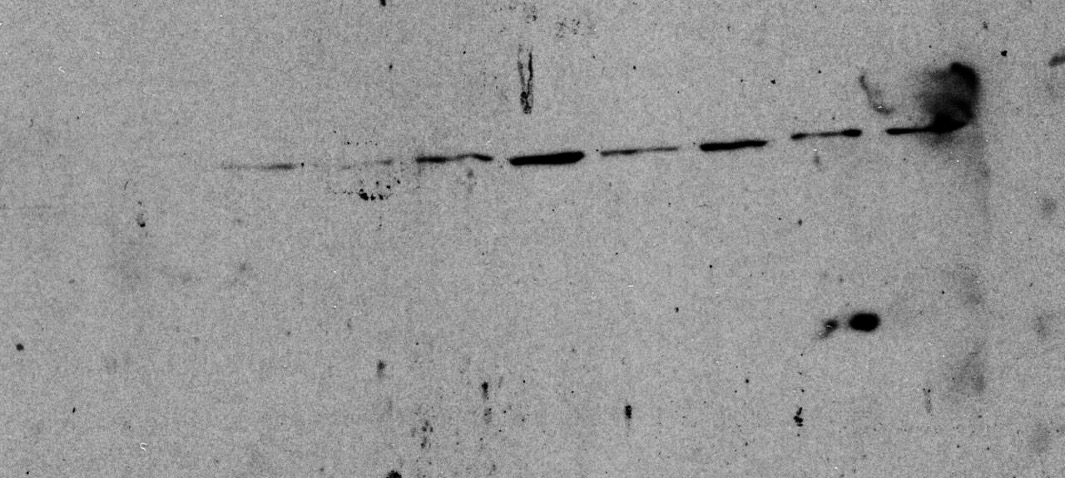

Supplement: Figure 11—figure supplement 4—source data 1. [file elife-79620-fig11-figsupp4-data1.zip › Figure 11 figure supplement 4 source data 1/bactin.jpg]

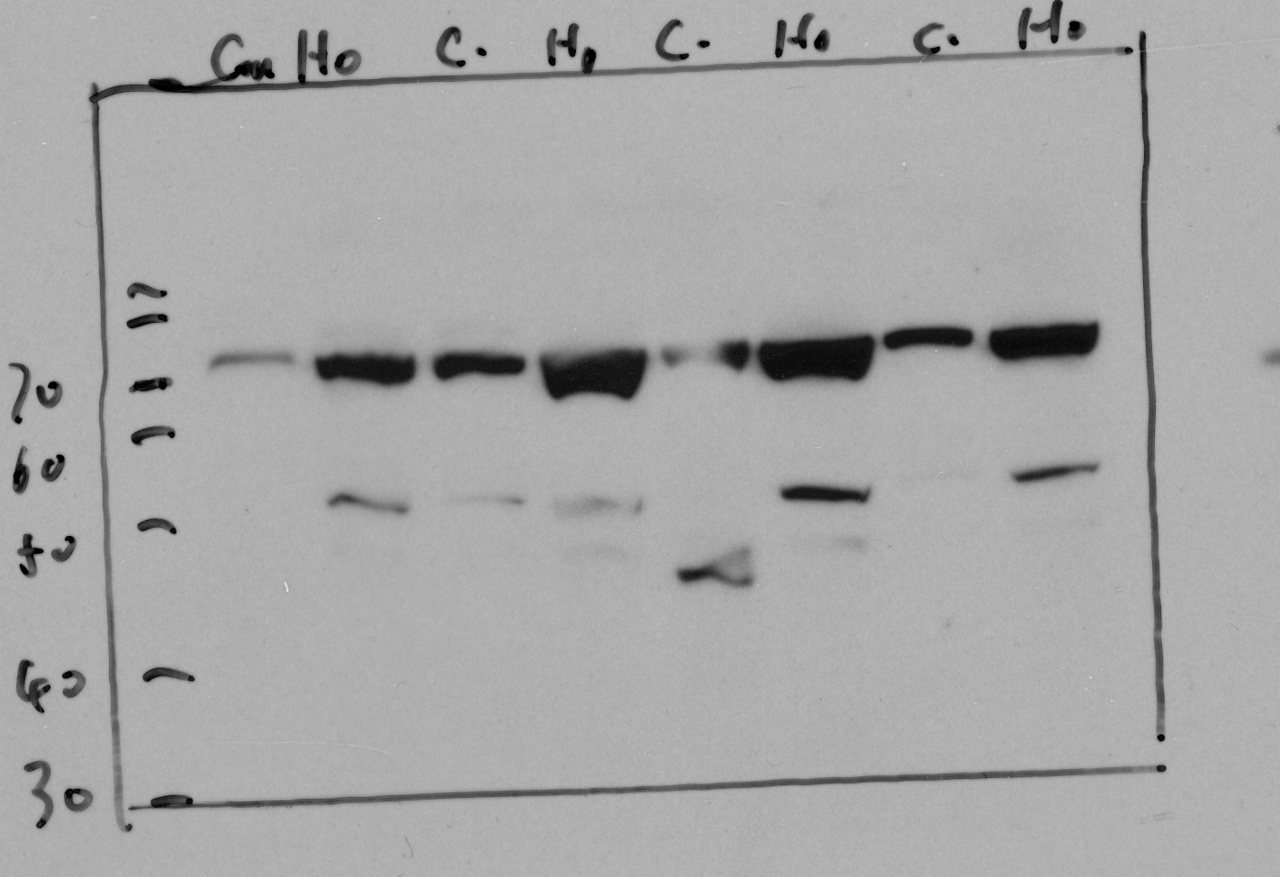

Supplement: Figure 11—figure supplement 4—source data 1. [file elife-79620-fig11-figsupp4-data1.zip › Figure 11 figure supplement 4 source data 1/Figure 11 figure supplement 4 data source 1.jpg]

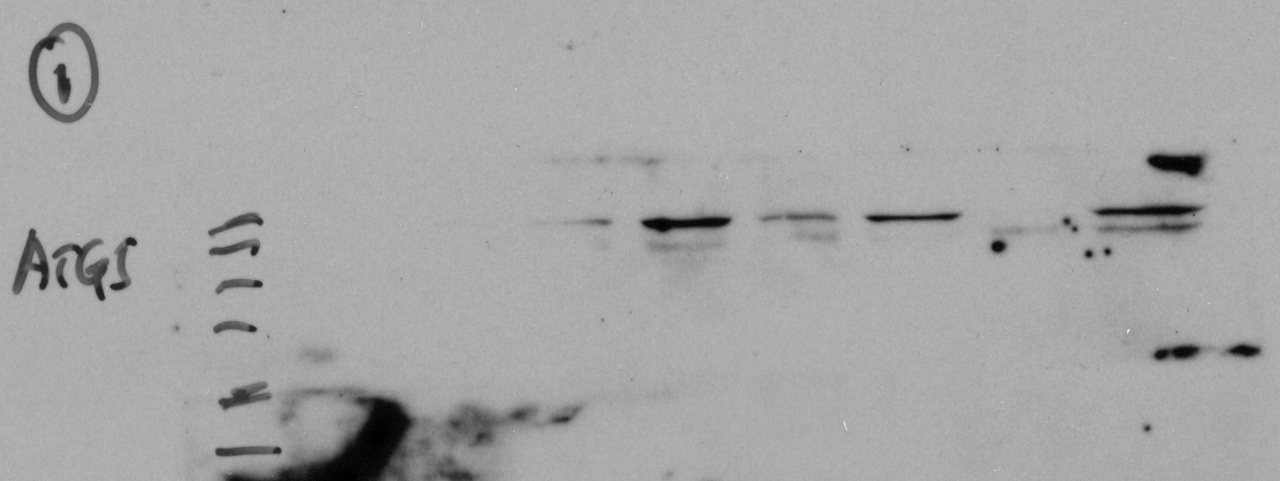

Supplement: Figure 11—figure supplement 4—source data 1. [file elife-79620-fig11-figsupp4-data1.zip › Figure 11 figure supplement 4 source data 1/Figure 11 figure supplement 4 data source 2.jpg]

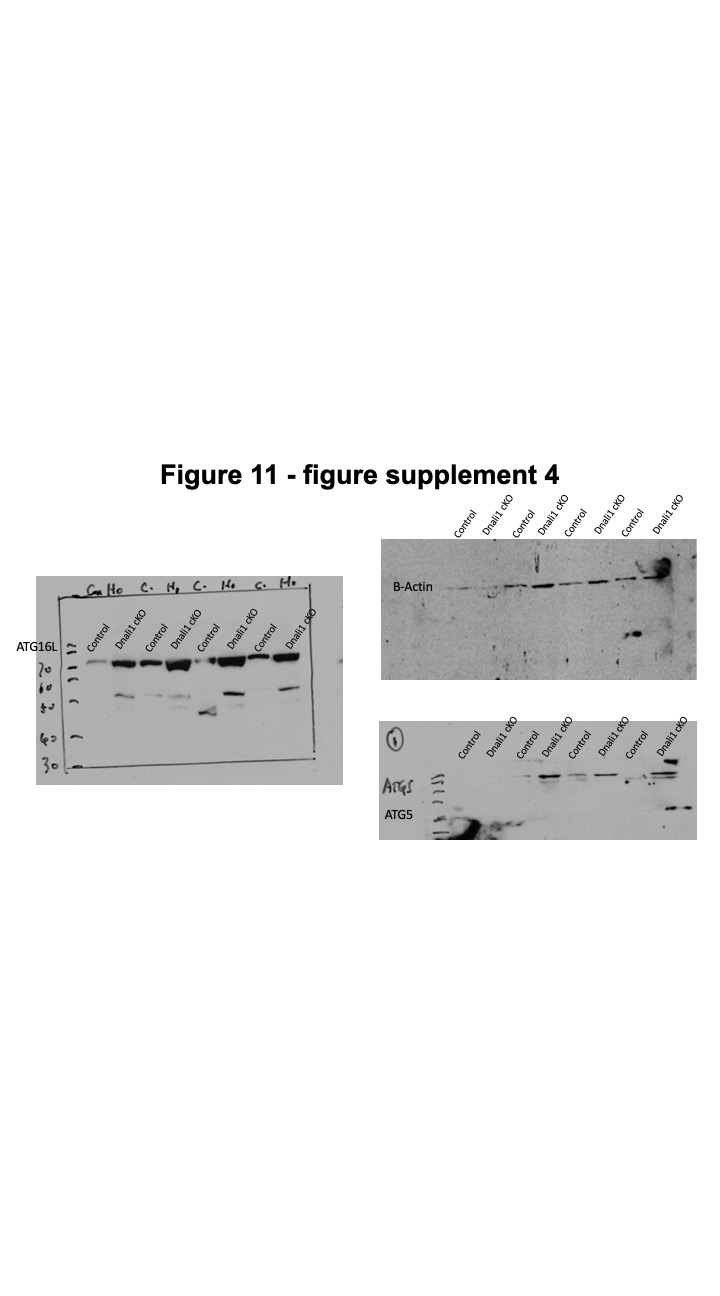

Supplement: Figure 11—figure supplement 4—source data 1. [file elife-79620-fig11-figsupp4-data1.zip › Figure 11 figure supplement 4 source data 1/Figure 11 - figure supplement 4 - source data 1/Figure 11 - figure supplement 4 - source data 1.jpeg]
